# Graduate Teaching Facilities in London Hospitals

**Published:** 1906-07-14

**Authors:** 


### GRADUATE TEACHING FACILITIES IN LONDON HOSPITALS.

### SEAMEN'S HOSPITAL (DREADNOUGHT), GREENWICH: THE LONDON SCHOOL OF TROPICAL MEDICINE.

THIS important centre of post-graduate education affords a valuable scope and abundant opportunities for the busy practitioner to burnish up his rusty knowledge in the short intervals at his disposal for such a purpose. Every facility is extended to him by the members of a particularly skilled staff of instructors. Every day during the past month most valuable instructions were given in nearly every practical department of medicinal and surgical science at the bedside of patients arriving from all parts of the world. The work is so synchronised that the practitioner gets full value for any time he may be disposed to allot to the school. The institution is reached in about 17 minutes from Charing Cross, Cannon Street, or London Bridge. A sketch of an afternoon's work at the London School of Clinical Medicine taken at random will afford an illustration of its methods and the advantages it offers to the general practitioner in search of new knowledge.

### CATARRH OF THE DUODENUM AND COMMON BILE DUCT.

Sir Dyce Duckworth, always a pleasure to hear and an instruction to see by the bedside of a patient, gave a demonstration of the cases in the wards under his charge. They included a case of chronic catarrhal jaundice, brought on from exposure at sea in a miserable craft in which the man was every moment expecting to go overboard. This was complicated by a rash. Sir Dyce mentioned that two rashes were connected with jaundice—xanthelasmus and prurigo; the first a scaly rash appearing on the inner eyelids, and on the extensor surfaces of the limbs; the other characterised by extreme irritation of the skin, almost as bad as scabies, and said to be due to bad bile pigment. This leads the patients to scratch and produce lesions of the skin. The patient in question had been seen by an authority [Sir Dyce considers it an insult to call a man a specialist; the proper term is an authority] on skin, Mr. Malcolm Morris. Erythematous rashes as a rule appear on the extensor surfaces; this one was present on both, and resembled to a large extent Pityriasis rubra, without, however, much braniness appearing. Pityriasis rubra, however, was not known to be connected with jaundice. The rash in this instance resembled a "plum-pudding rash." Sir Dyce has a practical way of teaching and amusingly demonstrated the agents with which the patient was being treated by asking the practitioners to say what was in the mixture. It turned out to be ammonium chloride gr. x. to xx., bicarbonate of soda gr. xv., and gentian. The ammonium chloride salt, Sir Dyce says, has a very stimulating effect on the liver, encouraging the production of bile and liquefying what has been already produced.

### CHRONIC PHTHISIS.

A case of chronic phthisis was shown in which no lesion could be found, in spite of frequent examination. On looking at the temperature range, however, Sir Dyce described it as "a useless, senseless temperature, one of the mysteries which are generally explained as tuberculosis—I don't know why!"

### NEURITIS.

This occurred in a Dane. On examining the legs, great muscle wasting was plain, almost like the legs in a case of beri-beri—knee jerks absent. On touching the peroneal nerve—the funny-bone of the leg—no pain was elicited. Great pain on pinching the gastrocnemius, just as happens when the legs of a secret gin-drinking woman are pinched. The foot had some power, but there was not much action of the peroneal muscles. Some degree of foot drop was present. No pain in the arms. The patient had taken quantities of beer, some spirits (Schnaps). "The prognosis here," said Sir Dyce, "is for chronicity. The pupils are sluggish, but not unequal. Rubbing, gentle massage, and the continuous battery are required. Don't give him iodide of potassium. Administer quinine, iron, fatty foods, and attend to his general nutrition. The temperature is irregular. Sometimes this can be put right simply by a change of environment. The atmosphere and not always the drains has to be looked to for an explanation."

### GASTRIC ULCER.

This was a relapse case in a Finn. The patient had vomited blood before he came to the hospital. The chief physical sign of gastric ulcer is pain in the mesial line for some reason, and going through to the back; the patient had had no sickness for a week, and was kept on sloppy milk diet. Sir Dyce recommended him to be fed now with a little bread and butter without crust, or a rusk soaked in milk, and simple custard pudding.

### MORBUS CORDIS.

A coloured native of Antigua had a displaced apex beat 1½ inches outside the nipple line showing hypertrophy of the left ventricle, a diffused impulse extending over the left margin of the sternum; the cardiac dullness measuring about five inches across. "Can you remember from your earlier studies how much it should be in a healthy adult man?" asked Sir Dyce from the group of practitioners around him. Answer: Less than four inches. "Only two inches," corrected Sir Dyce. "Two inches dullness for spleen, two for cardiac area, and four for hepatic dullness." A systolic murmur was present, accentuated pulmonary second sound, with extra pressure on the pulmonary artery. The liver was not enlarged, but engorged and swollen, and a little painful on pressure. The liver is the reservoir for a troubled engorged right side of the heart. The base of the right lung had crepitations. The patient was getting carbonate of ammonia, tincture of digitalis, tincture of nux vomica, and iodide of potassium.

Appendicitis with Subsequent Operation.

One of the most instructive cases was that of a young sailor who had arrived from Swansea. Sir Dyce pointed out a general rash over the patient, and remarked that, among other things, it might be due to a soap enema. "This," he said, "would not happen if a really pure soap like Vinolia were employed." If ordinary plain yellow soap were used, the paler it is the more likely it is to be pure. There was general distension of the abdomen, the right half appearing fuller than the left. The right rectus was on guard—fullness and hardness in the lower part of the abdomen. On deep pressure pain elicited over McBurney's point. Sir Dyce asked that a surgeon should see him, for, he remarked, "I am not going to be told by a surgeon if I had only sent in time the man would have lived."

The case was shortly afterwards seen by Mr. William Turner, surgeon to the hospital. His examination revealed that the man had been ill since he left Swansea, six days previously. A marked abdominal alteration was seen on the two sides—on the left side the anterior spine was evident; on the right it was obliterated. The pulse was small, irregular, jerky, not at all like the pulse of acute peritonitis. The facial aspect was important, and indicative of appendicitis. The pulse-rate altered as it went on—a very important diagnostic and prognostic point. On taking a deep breath, pain was felt under the liver, and the patient would not move the abdomen. The abdomen was tense, even on the left side; but quite hard on the right. The swelling occupied the right iliac fossa, extended to the middle line, and upwards beyond the umbilicus. "There is," he concluded, "no doubt about the diagnosis of the case, and the case should be operated on at once." It was necessary, he observed, to be careful with the manual examination, because by a very slight examination, if the swelling were associated with a perforated appendix or the abscess intraperitoneal, the slightest disturbance might send the pus round one of the corners of the adhesions into the peritoneal cavity, and set up a general peritonitis.

The Operation.

Mr. Turner operated on the patient. The appendix was gangrenous and in two pieces, and the tumour was full of septic pus. Two small fæcal accumulations as hard as pebbles occupied the lumen of the appendix, and were removed. The appendix had practically sloughed off. A large intraperitoneal abscess had been formed by the cæcum and intestine, not adherent at all to the peritoneum in front, but the cæcum was adherent to the side of the pelvic wall. The initial incision was made in the middle line like an ordinary laparotomy, and more than half a pint of pus escaped into a receiver. It was pointed out that in these cases the alteration of the pulse, when so marked as in this case, was of bad omen. When irregularity and inequality of pulse occur, before hardness and rigidity of the vessels in an abdominal trouble like appendicitis, it affords an imperative indication to operate. In this case another indication was found in the blood-count. The leucocytes on admission counted 14,000, on the day of operation 11,000, showing that the amount of toxæmia had been considerable to be able to destroy that number of leucocytes. The usual count for appendicitis with abscess, such as was present in the case here, would be about 21,000 leucocytes.

In spite of the critical conditions under which the operation was performed and the bad prognosis, the patient's temperature dropped to normal the next morning. The pulse recovered itself, and the general condition five days afterwards was excellent, and the patient is in a fair way for a speedy recovery.

### IN THE THROAT AND NOSE DEPARTMENT.

The visitors had the privilege also of a clinic in an important special department of practical work by Dr. St. Clair Thompson, who has recently made some important contributions to the literature and the practical scope of operations on the throat and nose. His instruction is simple, engaging, and thorough. He indicated, for example, the usual wrong way of nose testing by pushing the nose on one side, and compared it with right way of placing the thumb gently over the nostril. The open mouth gave rise to pharyngitis, headache from want of oxygenation, chronic winter cough, bronchitis, and a host of other troubles.

When examining the patient, Dr. Thompson observed that he should be placed as near as possible to the lamp, which should be exactly at the height of the patient's ear. Given a good light and with the examiner's eye about eight or nine inches distant from the patient, half the difficulty was achieved. The patient's nose should be examined before inserting the speculum. Ulcers or dislocations of the septum, for example, or any growth at the nares, may cause unnecessary pain. Thudicum's speculum should be used in preference to the old Voltolini's, and the various passages examined by tilting or lowering the head. With this speculum, which should be kept in check, the operator's right hand is free to use an instrument. The nose should be pulled up gently to see the nasal floor, because that is lower than the vestibule. On examining the pharynx an indication of mouth breathing was seen in the congestion of the soft palate ordinarily a little more pink than the hard palate. The patient had also a good crop of adenoids. In children about six or eight, on looking at the back of the throat, one often sees a large thing like an oyster sailing down the back of the throat. That is indicative of adenoids without further examination.

A towel placed over the patient's head is useful in digital examination for adenoids in preventing contamination and keeping the patient from starting upwards. The examining finger should not be passed directly backwards, but downwards towards the base of the tongue, then turned upwards forcibly and quickly into the middle line. The beginner should feel the septum, and instead of coming down over a fine surface a wormy feeling is imparted to the finger.

The patient obviously was suffering from tonsillitis and adenoids, besides having a deviating septum. It was arranged to remove the tonsils and the adenoids, and afterwards to cure the septal deviation. In this connection it may be noted that Dr. St. Clair Thompson recently read at the Congress at Lisbon a report on 30 operations on submucous excision of deviations and spurs of the nasal septum, an operation apparently very suitable for persons above the age of 17.

### Some Simple Remarks on Multiple Nasal Polypi.

Dr. St. Clair Thompson showed a young girl suffering from this derangement. Polypii had been removed frequently for the past five years from both sides of the nose. The nose was widened and tender at the part corresponding with the nasal bones outside and overlying the ethmoid. The discharge was copious, chiefly on the left side. There was no toothache. Recurrent polypi are usually associated with nasal suppuration, and most likely originate in the ethmoid cells, the frontal or maxillary being sometimes implicated. The sphenoidal sinus does not give rise to polypii. When removed they seem to grow quicker than ever unless the radical cure can be done. The first thing is to make sure of the diagnosis. One could make sure from the tenderness in this case that the ethmoid was affected. Some time was required to show whether the other cavities were also affected. To be done thoroughly, the operation should be done under chloroform. Dr. St. Clair Thompson has just published an account of three cases in which spontaneous evacuation of fronto ethmoidal sinusitis took place in the region of the orbit. If the sinus suppuration be of long standing, and the abscess due to retention or to infection, say, from multiple polypii, a radical operation was certainly called for.

